# The FGF2‐induced tanycyte proliferation involves a connexin 43 hemichannel/purinergic‐dependent pathway

**DOI:** 10.1111/jnc.15188

**Published:** 2020-10-19

**Authors:** Antonia Recabal, Paola Fernández, Sergio López, María J. Barahona, Patricio Ordenes, Alejandra Palma, Roberto Elizondo‐Vega, Carlos Farkas, Amparo Uribe, Teresa Caprile, Juan C. Sáez, María A. García‐Robles

**Affiliations:** ^1^ Departamento de Biología Celular Universidad de Concepción Concepción Chile; ^2^ Departamento de Fisiología Facultad de Ciencias Biológicas Pontificia Universidad Católica de Chile Santiago; ^3^ Research Institute in Oncology and Hematology Winnipeg Manitoba Canada; ^4^ Instituto de Neurociencias Centro Interdisciplinario de Neurociencias de Valparaíso Universidad de Valparaíso Valparaíso Chile; ^5^ Present address: Department of Physiology and Pathophysiology Max Rady College of Medicine University of Manitoba Winnipeg Manitoba Canada

**Keywords:** cell biology, Tanycyte, gliogenesis, purinergic receptors, signal transductions, tanycyte

## Abstract

In the adult hypothalamus, the neuronal precursor role is attributed to the radial glia‐like cells that line the third‐ventricle (3V) wall called tanycytes. Under nutritional cues, including hypercaloric diets, tanycytes proliferate and differentiate into mature neurons that moderate body weight, suggesting that hypothalamic neurogenesis is an adaptive mechanism in response to metabolic changes. Previous studies have shown that the tanycyte glucosensing mechanism depends on connexin‐43 hemichannels (Cx43 HCs), purine release, and increased intracellular free calcium ion concentration [(Ca^2+^)_i_] mediated by purinergic P2Y receptors. Since, Fibroblast Growth Factor 2 (FGF2) causes similar purinergic events in other cell types, we hypothesize that this pathway can be also activated by FGF2 in tanycytes to promote their proliferation. Here, we used bromodeoxyuridine (BrdU) incorporation to evaluate if FGF2‐induced tanycyte cell division is sensitive to Cx43 HC inhibition in vitro and in vivo. Immunocytochemical analyses showed that cultured tanycytes maintain the expression of in situ markers. After FGF2 exposure, tanycytic Cx43 HCs opened, enabling release of ATP to the extracellular milieu. Moreover, application of external ATP was enough to induce their cell division, which could be suppressed by Cx43 HC or P2Y1‐receptor inhibitors. Similarly, in vivo experiments performed on rats by continuous infusion of FGF2 and a Cx43 HC inhibitor into the 3V, demonstrated that FGF2‐induced β‐tanycyte proliferation is sensitive to Cx43 HC blockade. Thus, FGF2 induced Cx43 HC opening, triggered purinergic signaling, and increased β‐tanycytes proliferation, highlighting some of the molecular mechanisms involved in the cell division response of tanycyte.

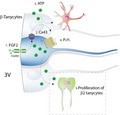

This article has an Editorial Highlight see https://doi.org/10.1111/jnc.15218.

## Graphical abstract

Fibroblast growth factor 2 (FGF2) accelerates basal‐tanycyte cell division, which is sensitive to Cx43 hemichannel inhibition in vitro and in vivo. In primary cultures of tanycyte, the mitogen FGF2 acts over FGF1R inducing Cx43 hemichannel opening and ATP release. Extracellular ATP induces tanycytic division through P2Y1 receptors. In vivo infusion of FGF2 with a Cx43 hemichannel inhibitor into the 3V showed less tanycytes in mitosis. These findings contribute to elucidate the molecular mechanisms involved in tanycyte proliferation.

**Abbreviations**
3V: third ventricle
[(Ca^2+^)]_i_: intracellular free calcium ion concentration
ARC: arcuate nucleus
ATP: adenosine triphosphate
BrdU: bromodeoxyuridine
CSF: cerebrospinal fluid
Cx43 HCs: connexin 43 hemichannels
Cx43: connexin 43
ENTPDase2: ecto‐nucleoside triphosphate diphosphohydrolase 2
ERK1/2: Extracellular signal‐regulated protein kinases 1 and 2
Etd^+^: ethidium bromide
FGF1: fibroblast Growth Factor 1
FGF2: fibroblast Growth Factor 2
FGFR1: fibroblast growth factor receptor 1
GABA: gamma‐aminobutyric acid
La^3+^: lanthanum ion
ME: median eminence
MRS2179: N6‐methyl‐2'‐deoxyadenosine‐3',5'‐bisphosphate
NPs: neuronal precursors
NPY: neuropeptide Y
Panx1: Pannexin1
POMC: pro‐opiomelanocortin
RRID: Research Resource Identifier
Sox2: sex determining region Y‐box 2
SVZ: subventricular zone
WGA: wheat germ agglutinin

## 1 INTRODUCTION

Tanycytes are specialized hypothalamic ependymal cells lining the lateral walls and floor of the third ventricle (3V) and are classified as reminiscent radial glia as well owing to their highly polarized morphology (Rodríguez et al., 2005). Their apical poles contact the cerebrospinal fluid (CSF), while some of their basal extensions project to the circumventricular organ median eminence (ME) or different hypothalamic nuclei, such as the arcuate nucleus (ARC) (Flament‐Durand & Brion, 1985), where the neurons responsible for energy balance and feeding behavior are located. Moreover, tanycytes represent a pool of neuronal precursor cells that proliferate and differentiate into functional orexigenic and anorexigenic neurons (Haan et al., 2013; Hajihosseini et al., 2008; Robins et al., 2013; Xu et al., 2005) after dietary exposure to high fat (Bless et al., 2016; Lee & Blackshaw, 2012). This early response adds new players to the neuronal network regulating feeding behavior and restoring energy balance (Gouaze et al., 2013; Kokoeva et al., 2005), prior to the pre‐obesity and pre‐diabetes activated inflammatory microenvironment (Li et al, 2012; Moraes et al., 2009). Furthermore, it has been suggested that tanycytes act as neuro‐modulating cells, regulating the availability and access of satiety‐ and hunger‐inducing hormones from peripheral tissue to ARC neurons (Balland et al., 2014; Collden et al., 2015; Langlet et al., 2013; Prevot, 2002). In addition, they express the molecular machinery for detecting nutrients, such as glucose, and for signaling to the adjacent neurons (Barahona et al., 2018; Cortés‐Campos et al., 2011; Elizondo‐Vega et al., 2016; García et al., 2003; Millán et al., 2010), which include connexin 43 hemichannels (Cx43 HCs) (Orellana et al., 2012). The role of Cx43 HCs and purinergic signaling on the glucosensing potential of tanycytes has been demonstrated in living hypothalamic slices (Frayling et al., 2011), while the specific mechanism through which a rapid (within min) increase in (Ca^2+^)_i_ occurs has been examined in primary cultures of tanycytes using pharmacological approaches. It sequentially consists of glucose transport and glycolytic metabolism, the controlled ATP release to the extracellular milieu mediated mainly by Cx43 HCs, and subsequent activation of P2Y receptors (Orellana et al., 2012). In HeLa cells, spinal astrocytes and glioma cells, the activity of Cx43 HCs have been shown to increase slowly (within hours) after a mitogen stimulus, such as Fibroblast Grow Factor 1 (Garré et al., 2010; Schalper et al., 2008) and 2 (De Vuyst et al., 2007) (FGF1 and 2, respectively), also triggering ATP release and purinergic signaling activation. FGF2 has been implicated in adult neurogenesis in the classic neurogenic niches such as the subventricular zone (SVZ) and the subgranular zone (SGZ) of the hippocampal dentate gyrus (Woodbury & Ikezu, 2014). Indeed, a combination of FGF2 and EGF, has been widely used for the maintenance and proliferation of neurospheres from different neurogenic niches (Kano et al., 2019; Furube et al., 2020). Extensive in situ evidence informed the expression of FGF2 receptor, FGFR1, in the ventral tanycyte domain (Kaminskas et al., 2019; Samms et al., 2015) and highlighted its importance in the control of body weight and food intake (Samms et al., 2015). Moreover, tanycyte cells divide under FGF2 stimulus (Robins et al., 2013; Xu et al., 2005). Thus, it is feasible to hypothesize that FGF2 activates a purineric signaling in which proliferation of tanycytes could result from a combined FGF2/Cx43 HCs/purinergic interaction.

Caloric restriction and a high‐fat diet have been identified as metabolic stimuli that influence the proliferation of adult hypothalamic neuronal precursors (NPs), but knowledge of the underlaying mechanisms are currently scarce (Bless et al., 2016; Chaker et al., 2016; Nascimento et al., 2016). It is likely that, under hypercaloric stimuli, tanycytes signal through multiple molecular pathways that may be redundant with those involved in metabolites detection. Here we show that in vitro tanycytes trigger Cx43 HCs opening and ATP release after long‐term exposure to FGF2 mitogen. In addition, a moderate increase in ATP concentration in the extracellular media was enough to induce cell division that can be suppressed by Cx43 HC and P2Y1 receptor inhibition. Moreover, in vivo experiments performed by directly infusion of a Cx43 HC blocker to the 3V showed a decline in the FGF2‐induced ventral tanycyte proliferation. These results suggest an essential role of Cx43 HCs and purinergic signaling in tanycyte self‐renewal.

## 2 MATERIALS AND METHODS

### 2.1 Ethics statement

All the studies performed on rats were approved and reviewed by the Animal Ethics Committee of the National Commission of Chile for Scientific and Technological studies (CONICYT, Fondecyt N°1180871), by the ethics committee of the Faculty of Biological Sciences and by the committee on Ethics, Care and Use of Animals of the University of Concepción, Chile. The animals were handled according to the guidelines of the National Institute of Health for the care and use of animals, USA. Male Sprague–Dawley rats (120–280 g) obtained from Charles River original source (RRID _10395233) were housed on a 12 hr light/dark cycle with food and water ad libitum. Each cage housed at least two adult animals and did not exceed the five animals. The hypothalamus of only one female adult rat was used for immunohistochemistry. Pregnant rats were housed in pairs. Each T25 flask of primary cell culture was performed of at least three and up to six hypothalami from post‐natal (PN) 1 rat Sprague–Dawley pups. In vivo studies were performed using a total of 15 Sprague Dawley adult male rats, weighing 120–180 g. Experimental procedure was incorporated in Figure 7. Animals were arbitrarily assigned to experimental groups; no randomization was performed. The study was not pre‐registered; all relevant information is provided in the manuscript and custom‐made materials will be provided upon request.

### 2.2 Primary cultures of PN1 tanycytes

Primary rat tanycyte cultures were performed according to the method described previously (García et al., 2003; Orellana et al., 2012). PN 1 rats were quickly decapitated, the brain was removed, and the 3V boundaries were dissected on ice. The samples were incubated with 0.25% trypsin–0.2% EDTA (w/v) (Thermo Fisher Scientific Inc.; Cat# 25200114) for 20 min at 37°C, before being transferred to MEM culture medium (Thermo Fisher Scientific Inc.; Cat# 61100087), supplemented with 10% fetal bovine serum (FBS) (Thermo Fisher Scientific Inc.; Cat# 12484028), 2 mM L‐glutamine, 100 U/mL penicillin, and 100 mg/ml streptomycin (Thermo Fisher Scientific Inc.; Cat# 15140163). The samples were disaggregated, and the cells were seeded in T25 culture flasks covered with 0.2 mg/ml poly‐l‐lysine (Sigma‐Aldrich; Cat# P6407) at a density of 3 million cells per flask. The cells were kept in the same bottle for 2 weeks, and the medium was renewed every other day. For subsequent experiments, monolayer‐grown tanycytes were rinsed twice with 0.1 M phosphate buffer (PBS; in mM: 137 NaCl, 2.7 KCl, 10 Na_2_HPO_4,_ and 2 KH_2_PO_4_) at pH 7.4 and treated with 0.25% trypsin–0.2% EDTA (w/v) for 3 min at 37°C. The cells were disaggregated and reseeded in 6, 12, and 24‐well plates, previously covered with 0.01% poly‐l‐lysine (w/v), at a cell density of 500,000, 250,000, and 800 cells per well, respectively. Only cells in passage one were used. Before all the experiments, tanycytes were cultivated for 24  hr in medium without FBS to prevent inhibition of ATP release and activation of purinergic signaling (Lin et al., 2007).

### 2.3 Total RNA extraction

Total RNA was obtained from samples of total brain, hypothalamus, striated muscle, and primary cultures of tanycytes. The RNA was extracted according to the guanidinum thiocyanate–phenol–chloroform method, homogenizing the samples in 500 ml Trizol® (Life Technology; Cat# 15596018) for 10 min and incubating them 5 min at room temperature (20ºC). Then, the samples were treated with 200 ml chloroform, vigorously shaken for 15 s, and incubated at room temperature (20ºC) for 3 min. The samples were centrifuged at 12,000 g for 15 min at 4°C to separate the phases. The aqueous phase was recovered, and 300 µl of isopropanol was added to each sample, incubated for 10 min at room temperature (20ºC), and centrifuged at 12,000 g for 5 min at 4°C. The supernatant was discarded, and the pellet was washed twice with 70% ethanol, spinning at 12,000 g for 10 min each time. Finally, the pellet was resuspended in 10 µl of RNase‐free water and quantified by measuring its absorbance at 260 nm and its purity according to the 260/280 ratio.

### 2.4 Reverse transcription (RT) of total RNA

DNA synthesis was performed using RevertAid® H Minus M‐MuLV Reverse Transcriptase Enzyme (Thermo Fisher Scientific Inc.; Cat# EP0451). Prior to synthesis, 2 µg of the total RNA samples were treated with DNase (Fisher Scientific Inc.; Cat# AM2238). For a final volume of 20 µl, the above mixture was incubated with 0.5 µg of oligo‐dT, denatured at 70°C for 5 min, and placed on ice for 2 min. Subsequently, the transcription buffer consisting of 50 mM Tris‐HCl (in mM: 50 KCl, 4 MgCl_2_, 10 DTT) at pH 8.3, the mixture of dNTPs (1 mM each), and 20 U of the ribonuclease inhibitor (Thermo Fisher Scientific Inc.; Cat# 10,777,019) were added, incubating for 5 min at 37°C. Next, 200 U of RevertAid® H Minus M‐MuLV (Thermo Fisher Scientific Inc.; Cat# EP0451) reverse transcriptase enzyme was added and incubated for 1 hr at 42°C followed by incubation at 70°C for 10 min. Negative controls for sample amplification were treated with the same protocol, but without adding oligo‐dT or reverse transcriptase enzyme to the mixture.

### 2.5 Amplification of cDNA by PCR

Amplification of cDNA was performed in an Eppendorf® Mastercycler® Nexus Thermal Cycler (Merck KGaA, Darmstadt, Germany) in a mixture of 10 mM Tris‐HCl at pH 8.8 containing 50 mM KCl, 1.5 mM MgCl_2_, 0.2 mM of each dNTP, 0.2 μM of each set of specific primers (Table 1), 0.31 U Taq DNA polymerase (Thermo Fisher Scientific Inc.; Cat# 10342053), and 1 μL of the reverse transcription product, in a final volume of 11.5 μL. The incubation program consisted of 95°C for 5 min, followed by 35 cycles of: denaturation 95°C for 30 s, annealing 55°C for 30 s, and 72°C for 30–40 s, and a final extension of 72°C for 7 min. The cDNAs synthesis was tested using specific β‐actin primers.

**TABLE 1:** Set of primers used for RT‐PCR

Gene	Sense (5'−3')	Antisense (5'−3')
P2Y1	ATTTGAGGGCACGGCTGGATTT	TGTGTCTCCGTTCTGCTTGAACTC
P2Y2	CTCTGTCATGCTGGGTCTGCTTTT	GCAACTGAGGTCAAGTGATCGGAA
P2Y4	TTGATCACCCTGGTCTGCTATGGA	CACATGACAGTCAGCTTGCAACAG
FGF1	CCTGACCGAGAGGTTCAATCTG	TCATTTGGTGTCTGCGAGCC
FGF2	GCAGAAGAGAGAGGAGTTGTGTCC	TGCCCAGTTCGTTTCAGTGC
FGFR1	CCTCTACGCTTGTGTGACCAACAG	TGCTCCCATACTCGTTCTCCAC
Cx43	TGGGTACAAGCTGGTTACTGGTGA	TGGCTAATGGCTGGAGTTCATGTC
β‐actin	GCTGCTCGTCGACAACGGCTC	CAAACATGATCTGGGTCATCTTCTC

### 2.6 Agarose gel electrophoresis

The separation of DNA fragments was performed using 1% agarose gels. The buffer used for the electrophoresis was TAE (Tris‐acetic acid EDTA; 40 mM Tris‐HCl, 30 mM acetic acid and 1 mM EDTA; pH 7.6). Agarose gels were prepared with TAE containing 0.5 µg/ml ethidium bromide (Genesse Scientific, Inc., San Diego, California, USA; Cat# 20‐276). A 100‐bp DNA ladder (GeneRuler, Thermo Scientific; Cat# SM0242) was used as the molecular weight marker. Transilluminator and photodocumentation kit (VilberLourmat PMM9A, France) were used to visualize the bands.

### 2.7 Ethidium uptake and fluorescence imaging

Tanycytes were transferred to poly‐l‐lysine ‐covered coverslips, grown to at least 80% confluency in six‐well plates and treated for 7 hr at 37°C with a final concentration of 20 ng/ml FGF2 (Sigma Aldrich; Cat# SRP4037) conjugated with heparin 10 IU/mL to maintain a ratio of 5 IU heparin for every 10 ng of FGF2, as it has been described by Schalper et al., (2008). Gap27 (Genscript, Piscataway; (SRPTEKTIFFI, second extracellular loop domain of Cx43), a selective Cx43 HC inhibitor, was added at a final concentration of 200 µM at the same time as FGF2. All compounds were added in the MEM culture medium supplemented with L‐glutamine, penicillin, and streptomycin, but without FBS. Each supplementation had a half‐hour lag (time that each registration lasted), starting at 9 a.m. and recording at 5 p.m. For fluorescence detection, cells were washed twice with 0.1 M PBS, pH 7.4, before applying the registration solution (154 mM NaCl, 5.4 mM KCl, 2.3 mM CaCl_2_, and 5 mM HEPES, pH 7.4), containing 5 µM ethidium bromide (Etd^+^, Sigma Aldrich; Cat# E7637). Cells on the coverslips were mounted under a live cell microscope (Nikon, Eclipse Ti‐FL model, Japan) and were recorded every 30 s with a 40x objective. At least 10 cell nuclei per coverslip were defined as regions of interest (ROIs), and the average change in their fluorescence intensity was measured over time. After approximately 30 min, 200 µM lanthanum ion (La^3+^, LaCl3x7H2O, Sigma Aldrich; Cat# L4131), which is a non‐selective inhibitor of Cx HCs as a control of cell vitality, was added.

### 2.8 Extracellular ATP measurements

Tanycytes were seeded on 12‐well plates, previously covered with poly‐l‐lysine and cultured without FBS for 24 hr at 37°C, before being treated with a final concentration of 20 ng/ml FGF2, 10 IU/mL heparin, and/or 200 µM Gap27. The final volume in the culture medium was 300 µl for each well. All the experiments started at 10 a.m., and after 7 hr (5 p.m.), the concentration of ATP contained in 100 µM was measured through the luciferin/luciferase bioluminescence assay (ATP Bioluminescence Assay Kit CLS II, Sigma‐Aldrich; Cat# 11699695001). The amount of ATP in each sample was calculated from a standard curve, whose concentration range includes 10, 20, 40, 60, 80, and 100 pM. CPS (counts per second) values that exceeded the curve values were not considered. ATP concentration was normalized to the total protein concentration of its respective sample, using the Bradford reagent (Bio‐Rad Laboratories; Cat# 5000006EDU) and Optizen Pop spectrophotometer (Comercial Rafer SL, Zaragoza. Spain).

### 2.9 Immunofluorescence

For immunohistochemistry, the rats were anesthetized with 200 µl of intraperitoneal ketamine:xylazine (90 mg/kg‐10 mg/kg) until they exhibited no reflections. They were intravascularly perfused with 200 ml ice cold PBS and then 200 ml of 4% PFA. The cervical were dislocated, the brains were removed, the hypothalami were dissected and slices of 200 µm thickness were performed using vibratome (Leica VT1200, Wetzlar, Alemania). For immunocytochemistry, once treated, cells were washed once with 0.1 M PBS and fixed with 4% (w/v) paraformaldehyde dissolved in PBS for 30 min at room temperature (20ºC). The wheat germ agglutinin procedure was considered a pre‐fixation step that consisted of bathing the cells for 30 s with 1 µg/ml WGA (Sigma‐Aldrich; Cat# L9640) that was dissolved in cold PBS and then washed twice with the saline solution. The BrdU detection was performed with an additional step that allowed DNA denaturation through 1 M HCl at 45°C for 30 min, before blocking. The acid was neutralized by rinsing the slices or cells three times for 10 min with Tris phosphate buffer (84 mM Na_2_HPO_4_, 35 mM KH_2_PO_4_, 120 mM NaCl, 10 mM Tris, pH 7.8). The following primary antibodies dissolved in Tris phosphate with 0.1% Triton X‐100 were incubated overnight at room temperature (20ºC): sheep anti‐BrdU (1:2,000, Abcam; RRID AB_302659), rabbit anti‐Cx43 (1:200, BD Biosciences Franklin Lakes; RRID:AB_397473) previously described (Schalper et al., 2008), mouse anti‐Nestin (1:1,500, Abcam; RIDD AB_305313), mouse anti‐vimentin (1:400, DAKO; RIDD AB_2827759), goat anti‐SOX2 (1:400, Santa Cruz; RIDD AB_2286684), and rabbit anti‐WGA (1:1,000, Sigma Aldrich; RIDD AB_261669). The samples were washed three times with the Tris phosphate buffer and incubated for 2 hrwith the respective secondary antibodies bound to fluorophores and TOPRO (Thermo Fisher; Cat# T3605) in a final dilution of 1:200 and 1:1,000, respectively. Finally, the coverslips were mounted with FluoromontTM Aqueous Mounting Medium (Sigma Aldrich; Cat# F4680). The samples were analyzed by confocal microscopy (Confocal Spectral Microscope, model LSM780 NLO Zeiss, Centro de Microscopía Avanzada, CMA‐Bio Bio).

### 2.10 Protein immunodetection

Total protein extracts were obtained from samples of heart, liver, and primary cultures of tanycytes. The samples were homogenized in protease inhibitor (Thomas Scientific, ROCHE cOmplete™; Cat# 12352200) and sonicated three times on ice at 300 W in the case of tissues or resuspended in 40 µl of lysis buffer (0.5% Igepal CA030, 10 mM Hepes pH 7.9, 1 mM DTT, 100 mM NaCl, 0.5 mM PMSF) in the presence of protease and phosphatase inhibitors in the case of cells within the culture. Concentrations of the lysed proteins were measured by the Bradford technique, equaled to 25–50 µg and incubated for 1 min at 80°C with a protein loading buffer (62.5 mM Tris‐HCl pH 6.8, 2% SDS, 10% glycerol, 0.01% bromophenol blue) in the presence of 0.1 M DTT. Proteins were separated on a 1.5‐mm width 12% acrylamide denaturing gel (SDS‐PAGE). The samples were seeded next to the pre‐stained standard (Spectra Multicolor, Broad Range Protein Ladder, Thermo Scientific, Cat# 26623) in the gel, then separated at 80 V in a solution containing 25 mM Tris, 250 mM glycine, and 0.1% SDS. Proteins were subsequently transferred to an Immobilon‐P membrane (0.45 μm pore, Merck Millipore; Cat# HVLP04700) in transfer solution (25 mM Tris, 192 mM glycine, 20% methanol) for 2 hr at 250 mA. To verify correct transfer, the nitrocellulose membrane was stained with "Ponceau" red (Sigma Aldrich; Cat# P3504‐10G). Multiple washes were performed with TBS‐Tween (150 mM NaCl, 10 mM Tris, 0.05% Tween20), followed by blocking the membrane with 5% skim milk in TBS‐Tween for 1 hr. The membranes were next incubated with the primary antibody and subsequently with the secondary, both dissolved in 5% skim milk TBS‐Tween at 4°C overnight and at room temperature (20ºC) for 1 hr, respectively. The primary antibodies used were mouse anti‐Cx43 (dilution 1:1,000, BD Biosciences), rabbit anti‐lamina B1 (dilution 1:1,000, Abcam), and anti‐phospho ERK1/2 Thr202/Tyr204 (dilution 1:2,000, BioLegend; RRID:AB_2721734). The secondary antibodies used were peroxidase‐conjugated anti‐mouse and anti‐rabbit (1:1,000; Jackson ImmunoResearch Laboratories; Cat# 715‐035‐150; Cat#711‐035‐152). After secondary antibody incubation, three washes with TBS‐Tween were performed for 10 min. Finally, the membrane was exposed using a Western Lightening Plus‐ECL kit (Perkin Elmer; Cat# NEL103001EA) on the luminescent chemo and fluorescence imaging equipment (PXi, Syngene).

### 2.11 Incorporation of BrdU in cell cultures

Tanycytes were seeded on 8‐mm coverslips covered with poly‐l‐lysine in 24‐well plates. The cultures were incubated at 37°C for 26 hr with a final concentration of each compound (or the mixture of them): 20 ng/ml FGF2, 10 IU/mL heparin, 200 µM Gap27 and 10, 50, 100, or 200 µM ATP (Sigma Aldrich; Cat# A1852), 10 µM ATPɣS (Sigma Aldrich; Cat# 11162306001), and/or 10 µM MRS2179 (Sigma Aldrich; Cat# M3808). After 20 hr, the medium containing the same compounds was renewed, and BrdU was added in a final concentration of 10 µM for another 6 hr. The 6‐hr timepoint was selected since at 2 hr, few cells incorporate BrdU and at 14 hr, almost all were labeled (unpublished). After incubation, the cells were washed twice in 0.1 M PBS, pH 7.4 and fixed for 30 min with 4% paraformaldehyde for later immunocytochemical analysis.

### 2.12 Osmotic pumps preparation

The osmotic pumps (Alzet Model 1007D), with capacity of 100 µl and a continuous release rate of 0.5 µg/h for 7 days, were filled the day before implantation according to the manufacturer's manual. BrdU, FGF2, and Gap27 were dissolved in filtered CSF (1.25 mM NaH_2_PO_4_, 126 mM NaCl, 3 mM KCl, 2 mM MgCl_2_ 7H_2_O, 2 mM CaCl_2_ 6H_2_O, 26 mM NaHCO_3_, and 2 mM glucose, pH 7.4) at a final concentration of 1.5 µg/µl, 25 ng/µl, and 0.26 µg/µl, respectively. The stainless‐steel tube was fitted to a vinyl catheter with an internal diameter of 0.58 mm and a length of 3 cm and both were filled with the same solution. The osmotic pumps were equilibrated in sterile saline solution (0.9% w/v) at 37°C for 16 hr before being implanted. Following implantation, compounds were infused at a rate of 0.75 µg/h (BrdU), 0.0125 µg/h (FGF2), and 0.13 µg/h (Gap27).

### 2.13 Stereotaxic cannula implantation

The cannulas were implanted by 3V stereotaxis according to the protocol shown in Figure 7. Rats were anesthetized with an intraperitoneal injection of the ketamine/xylazine mixture (90 mg/kg–10 mg/kg) and the fur was shaved over the head to expose the area where the incision was made. Rats were attached to the stereotaxic kit with ear bars that do not rupture the eardrum. The shaved skin was cleaned with clorhexydine, an incision was made with a scalpel, and a hole was drilled with a trephine to implant a guide cannula (28 gauge stainless steel; Plastics One), according to the following coordinates: ‐ 3.14 mm in the anterior–posterior axis of the bregma (confluence point of the sutures of the frontal and parietal bones), 0.0 medial–lateral of the medial sagittal sinus, 9.2 mm dorsal–ventral from the upper part of the skull. The cannula guide was previously connected to the 2 cm catheter attached to the perfusion pump and secured to the skull using 3/32 mm mounting screws and dental acrylic. Once the dental acrylic had dried, the osmotic pump was incorporated through the same incision with the help of forceps and faced with absorbable 5/0 HR15 synthetic sutures (Tagum). Animals received a subcutaneous injection of tramadol (2 mg/kg) and 1% ketoprofen (10 mg/ml). The rats were individually housed after surgery, although the infusion of the osmotic pump content was immediate. Rats were sacrificed 8 days after the implantation of the osmotic pump using an intraperitoneal injection of the ketamine/xylazine mixture (90 mg/kg–10 mg/kg), then they were vascularly perfused and cervically dislocated. The protocol was supervised by the ethics committee of the university.

### 2.14 Statistics and image processing

All values were calculated as the average over each culture or animal. Significant differences were determined using the Student's *t*‐test or one‐way ANOVA with Bonferroni's multiple comparison post hoc test, as indicated otherwise. The normality of data was carried out using Shapiro–Wilk with a confidence value of 95%. For extracellular ATP measurements, CP values that were not within the standardization curve were excluded. For in vitro BrdU incorporation analysis, the visual field of each sample was blindly defined only by nestin fluorescence. Then, the number of BrdU‐positive cells was quantified using Fiji (Image J) software by first determining a subjective threshold, in which all cell nuclei were detected and detached from each other, and then applying the “Analyze Particles” tool with the following settings: “size (micron2)” in the range of 0.25‐infinity and “circularity” with range 0.00–1.00. Statistical significance was defined by *p* < .05 using the Graphpad Prism 7.0 program. No randomization was performed to allocate cultures in the study. The quantification of in vivo BrdU‐positive hypothalamic cells was computationally and objectively performed using Imaris 9.1.0 software (Centro de Microscopía Avanzada, CMA‐Bio Bio). The following parameters were established: (1) tanycytes and ependymal cells were considered as those cells with a proximity of 20 µm from the ventricular border, (2) the program determined the number of positive BrdU nuclei as those with a diameter greater than 7 µm and less than 12 µm (in images with 20X magnification), and (3) the total volume of the tissue was considered, excluding the ventricular area. Values with a standard deviation higher to 50% were discarded, no blinding was performed. The minimum sample size (experimental n) was determined according to the recursive equation of experiments that do not use blocks (i.e. sex or age), considering only the number of treatments and a confidence value of 95%.

## 3 RESULTS

### 3.1 In vitro tanycytes retain the expression of undifferentiation markers, Cx43, purinergic receptors, FGFs and their receptor

**FIGURE 1:**
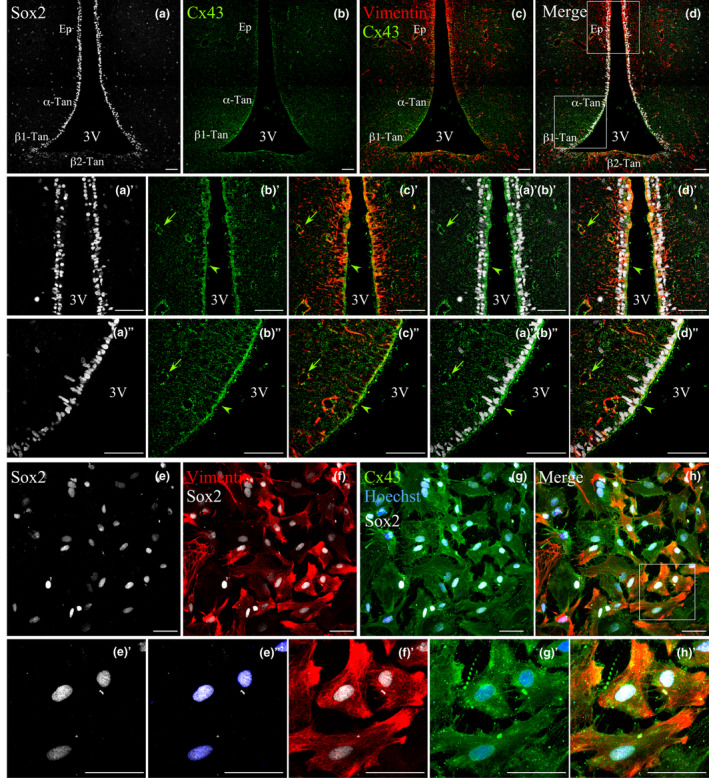
Connexin43 and undifferentiated markers are expressed by cultured tanycytes. (a–d) Representative confocal images of rat hypothalamus that show Sox2 (a‐a’’), Cx43 (b‐b’’) and vimentin (c‐c’’) reactivity. (d‐d’’) shows the three channels merged. The mentioned markers are present in α1 (a’‐d’), α2, and β1 (a’’‐d’’) tanycytes, which are specified in the magnification of the box in D. Arrowheads in (b‐b’’) point to Cx43 localization in the apical portion of tanycytes, while arrows in (b‐b’’) point its localization surrounding the blood vessels. (e–h) Immunocytochemistry of adherent primary cultures of tanycytes with antibodies against Sox2 (e‐e’), vimentin (f‐f’), Cx43 (g‐g’), and the merge of all channels (H‐H’). Hoechst was used for nuclei staining (e’, g‐g’). (e’, e’’‐h’) are augmented images of H box. 3V, third ventricle; Ep, ependymocytes; Tan, tanycytes. Scale bar: 50 µm. N: 1 animal and 3 primary culture

In mouse and rat hypothalamus, the overlapped expression of vimentin and nestin neuronal precursor markers is primarily restricted to tanycytes (Pellegrino et al., 2018). To evaluate the conservation of undifferentiation markers in primary cultures of tanycytes, the expression of Sox2, Cx43, nestin and/or vimentin (both showing the same reactivity in tanycytes, data not shown) was compared to their expression in vivo. Immunohistochemical assays of a hypothalamic frontal slice using antibodies against Sox2 (Figure 1a‐a’’), Cx43 (Figure 1b‐b’’) and vimentin (Figure 1c‐c’’) showed their joint expression in the lateral walls of the 3V (Figure 1d‐d’’), represented by tanycytic and ependymal cells at the ventral VZ and most dorsal portion, respectively as shown in Figure 1a–d. Sox2 was localized in the nuclei of ependymocytes (Figure 1a’), α1 close to the ependymocytes (Figure 1a’) and α2 close to the β1‐tanycytes (Figure 1a’’), whereas Cx43 was mainly detected in the apical portion of both α and β1‐tanycytes (arrowheads in Figure 1b’ and b’’, respectively). Cx43 was also present in β2‐tanycytes, but with minor intensity (Figure 1b). As previously observed, Cx43 was found surrounding the blood vessels of rat hypothalamic parenchyma (arrows in Figure 1b’‐b’’). Tanycyte primary cultures are usually performed under adherent conditions and consequently, they lose their polarized morphology (Figure 1e–h). Nevertheless, they retain the expression of nuclear Sox2 (Figure 1e‐e’’), vimentin (Figure 1f‐f’’) and Cx43 (Figure 1g‐g’).

**FIGURE 2:**
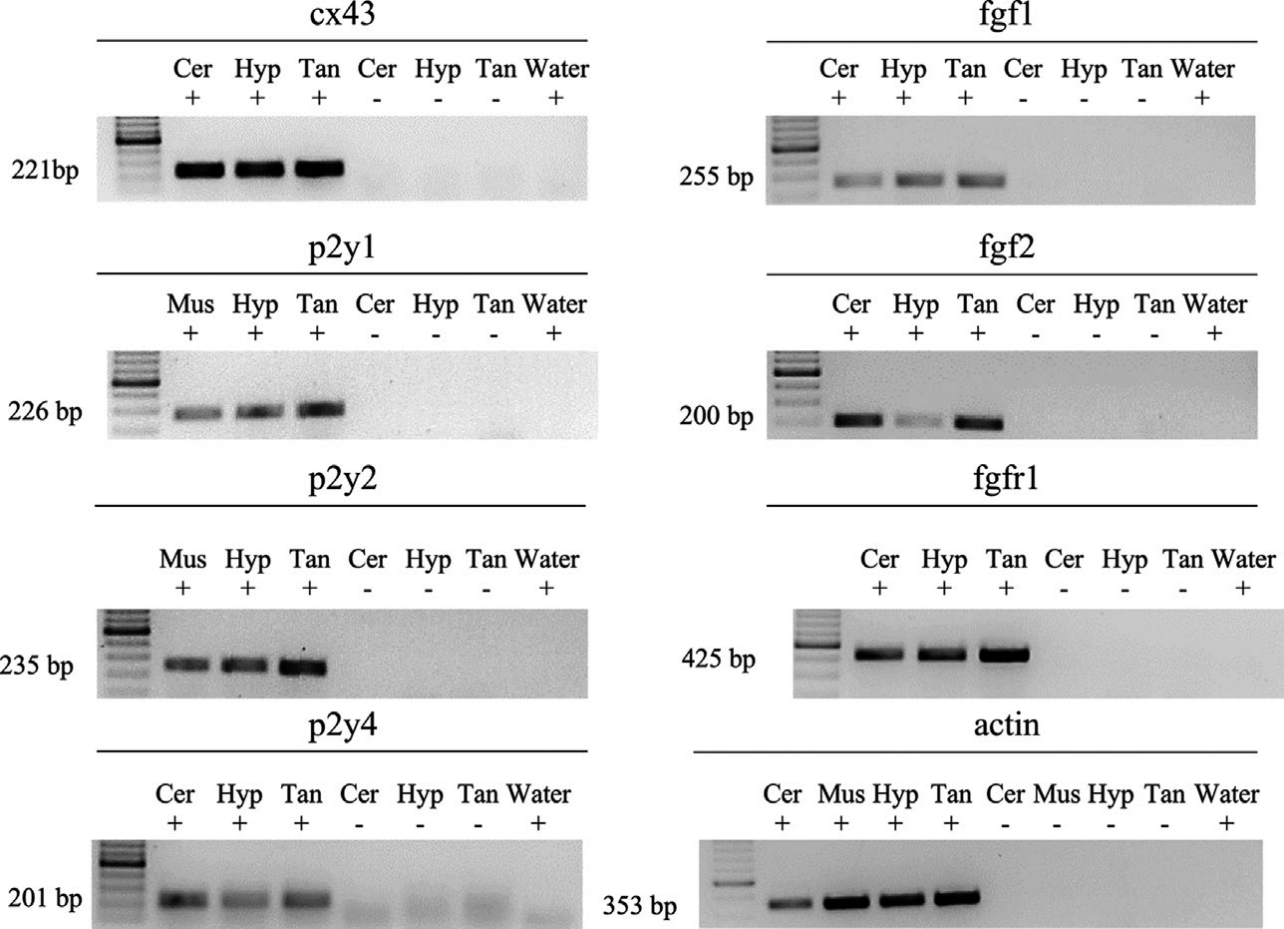
The relative mRNA amount of Cx43, purinergic signaling components, and FGF2 pathway in the primary culture of tanycytes. Specific primers were used in order to amplify a fragment indicative of the presence of the following mRNAs: cx43, p2y1, p2y2, and p2y4 purinergic receptors, fgf1, fgf2, and its receptor, fgfr1. Actin was used as a loading control. Total mRNAs were extracted from whole cerebral tissue (Cer), hypothalamus (Hyp), striated muscle (Mus), and primary culture of tanycytes (Tan). Retrotranscription was performed in the presence of MulV retrotranscriptase enzyme (positive signs), whereas the absence of the enzyme was used as a negative control (negative signs). The size of the amplicon is shown at the left side of each electrophoresis. The most intense band in the scale bar (first line) represents 500 bp. *N* = 2 primary culture

Considering that Cx43 forms hemichannels that are crucial to trigger purinergic signaling in response to glucose in tanycytes, we evaluated whether HCs Cx43 could also be activated and enhance purinergic signaling in response to mitogens, such as FGF2. At a first step, the expression of Cx43 and of the purinergic signaling receptors present in other neural precursors (NPs), as well as FGF receptor, which have been shown to increase the activity of Cx43 HCs opening, was evaluated transcriptionally. Conventional RT‐PCR analysis showed that Cx43, the metabotropic receptors p2y1, p2y2, and p2y4 (Lin et al., 2007), fgf1 (Garré et al., 2010; Schalper et al., 2008), fgf2 (De Vuyst et al., 2007) and its receptor, fgfr1, were expressed in both hypothalamic extracts (Hyp) and primary tanycytic cultures (Tan; Figure 2). Although the fgfr1 set of primers were designed to detect the long receptor isoform (containing the I Immunoglobulin‐like domain), the expression of the canonical FGF2 receptor isoform (IIIc) was already described by others (Kaminskas et al., 2019; Samms et al., 2015), specifically restricted to the ventral tanycyte subpopulation (Kaminskas et al., 2019).

### 3.2 Cx43 and P2Y1R are necessary for in vitro proliferation of tanycytes induced by FGF2

**FIGURE 3:**
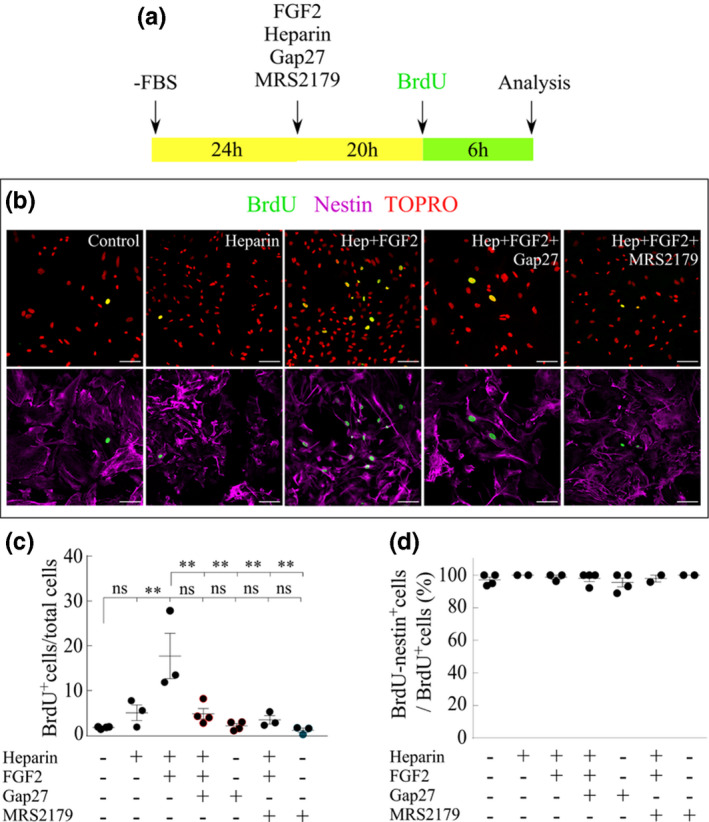
FGF2‐induced proliferation of cultured tanycytes is inhibited by Gap27 and MRS2179. (a) Timeline detailing the procedures performed to evaluate BrdU incorporation. (b) Immunofluorescence detection of BrdU (green) and nestin (magenta) immunoreactivity. TOPRO was used for nuclei staining (red). Scale bar: 100 µm. (c) Quantification of the BrdU positive cells (percentage) after the exposure to the FGF2 cofactor heparin, the mixture of FGF2/heparin, the mixture FGF2/heparin and blocker of either Cx43(Gap27) or P2Y1 receptor (MRS2179). (d) Quantification of the proliferative nestin‐positive cells over the total proliferative cells to assess the cell type‐specificity response to the stimuli. *N* = ≥12 replicates and three independent cultures per condition. One‐way ANOVA with Bonferroni post hoc. (**) *p* < .01, (ns) non‐significant. Data were represented as the average ± *SEM*

The importance of Cx43 and P2Y1 receptor in the proliferation response of tanycytes in culture was examined using Gap27 and MRS2179, respectively. While MRS2179 is a competitive P2Y1 receptor antagonist, Gap27 is a Cx43 mimetic peptide in its 204‐SRPTEKTIFFI‐214 amino acid sequence, which interacts with the second extracellular loop and blocks the activity of Cx HCs in min, but later on also prevents the pairing of two hemichannels, affecting the formation of gap junction channels (Abudara et al., 2014). The DNA replication that proceeds cellular proliferation of tanycytes was evaluated by BrdU incorporation assay. BrdU treatment was evaluated after 24 hr in culture without serum plus another 26 hr of different treatments. The compounds were dissolved in the culture medium and 6 hr before finishing, the medium was renewed and BrdU was added, as shown in Figure 3a. Quantification of the proportion of tanycytes that incorporated BrdU in the analyzed time frame was performed through immunocytochemistry with antibodies specific for BrdU and nestin. Nuclei were stained with TOPRO (representative images in Figure 3b), which showed that under normal conditions, only 1.7 ± 0.1% (mean ± SEM) of the cells underwent division (Figure 3c). Since FGFs require the presence of heparan sulfate proteoglycans to interact with their receptor (Itoh & Ornitz, 2004), a solution containing heparin was used to dilute and deliver FGF2 to the tanycytes (Schalper et al., 2008). The presence of 20 ng/ml of FGF2 together with its heparin cofactor, increased the proportion of proliferative cells in one order of magnitude, reaching a significant value of 17.7 ± 5.1% (Figure 3c). The heparin cofactor promoted a slight effect on the cell division rate, although not statistically significant, increasing proliferation to 5.0 ± 1.7% (Figure 3c). Interestingly, Gap27 (200 µM) and MRS2170 (10 µM) prevented the FGF2‐induced proliferation, reaching 4.8 ± 1.2% and 3.4 ± 0.9% respectively, which were significantly lower than that observed with FGF2, suggesting the importance of the Cx43/P2Y1R axis in the self‐renewal capacity of this cell type. It is important to note that the exposure to each inhibitor did not affect tanycyte cell division (2.1 ± 0.5% for Gap27 and 1.1 ± 0.5% for MRS2179; Figure 3c). Assuming that nestin is expressed only by tanycytes in primary cultures, the number of cells positive for both nestin and BrdU over the total number of BrdU‐positive cells was quantified to corroborate that the proliferation events observed concerned only tanycytes (Figure 3d). The specificity of the BrdU on tanycytes ranged between 95.5 ± 2.7 (for Gap27) and 100 ± 0% (for heparin and MRS2179; Figure 3d). These results demonstrated that the proliferation observed is restricted to tanycytes. The purity of the cultured tanycytes described here agreed with our previous reports using the same methodology, which have shown to contain more than 90% tanycytes (Orellana et al., 2012). Moreover, their intense reactivity to vimentin, Kir6.1, GLUT2, GK, MC1 and 4, but not to GFAP, MAP2, and βIII‐tubulin rule out the contamination with other hypothalamic cell types such as astrocytes and neurons and strongly suggest a highly prevalence of β‐type tanycytes (Cortés‐Campos et al., 2011; García et al., 2003; Millan et al., 2010; Orellana et al., 2012). Negative immunoreaction for GFAP in β tanycytes has been recently corroborated by Kano et al. (2019). Since the tanycyte cultures are mainly composed of β‐tanycytes, it is therefore possible to argue that the Cx43‐dependent FGF2‐proliferative response seen here applies to this cell subpopulation, without excluding a possible effect on α‐tanycytes.

### 3.3 FGF2 increases hemichannel activity and ATP release in cultured tanycytes

**FIGURE 4:**
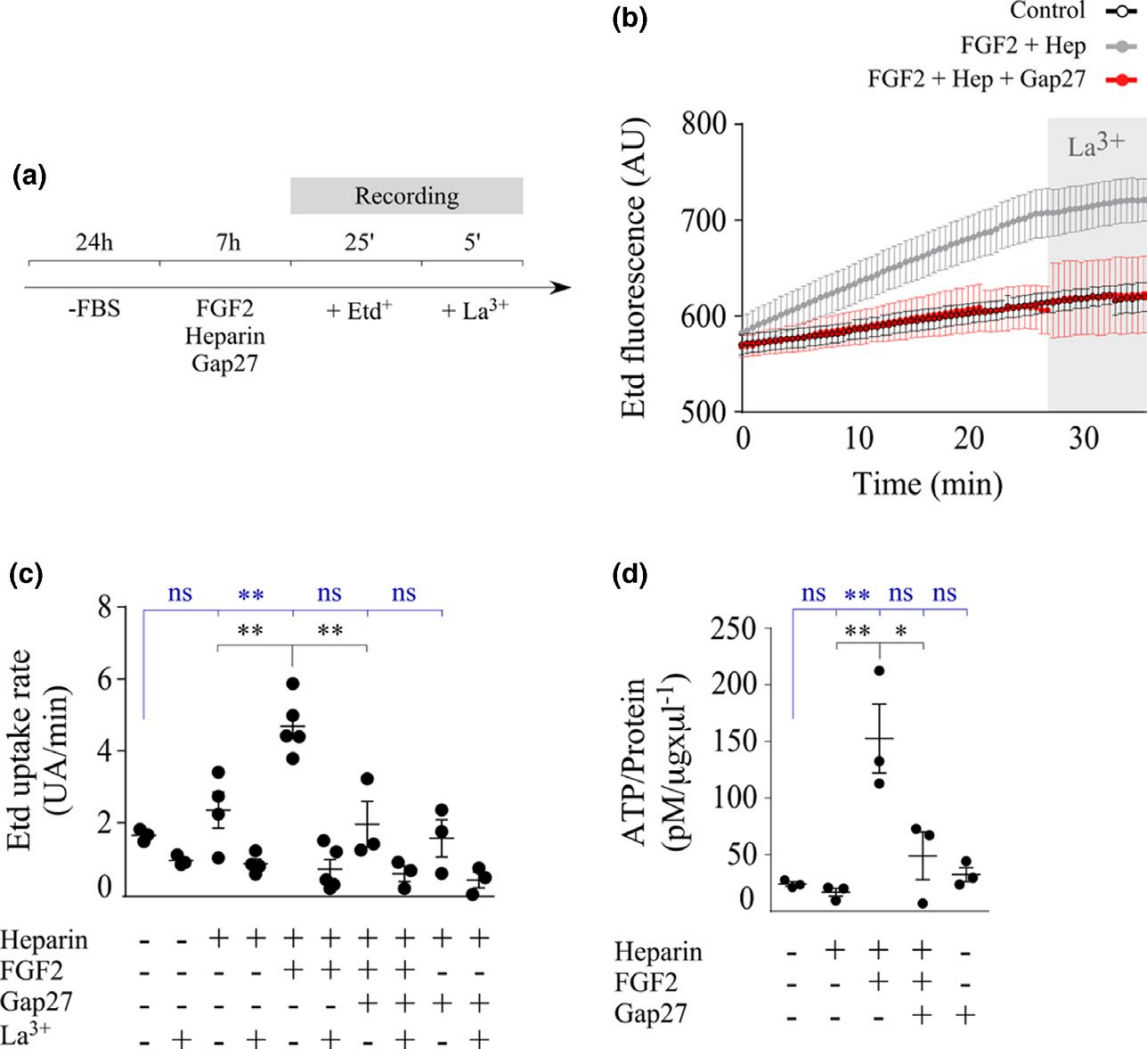
FGF2 induces ATP release via connexin hemichannels in cultured tanycytes. (a) Timeline detailing the procedures to measure ethidium (Etd^+^) uptake. (b and c) Etd^+^ uptake over time (b) and Etd^+^ uptake rate (c) after exposure of tanycytes to different treatments and quantified as arbitrary units of emitted nuclear fluorescence (AU) over time (min). For a clearer view, data in (b) show the Etd^+^ uptake average and *SEM* curves of only some conditions (control, FGF2/heparin, and FGF2/heparin/Gap27). The gray bar in (b) represents the moment when the Cx HCs inhibitor, lantanum ion (La^3+^), was added. The graph in (c) was originated from the slopes in (b). *N* = ≥10 nuclei per culture and three independent cultures per condition were analyzed. (d) Luciferin/Luciferase signal obtained in measurements of the amount of extracellular ATP (pM), normalized to the total protein content (µg/µl). *N* = ≥3 independent cultures per condition. One‐way ANOVA with Bonferroni post hoc. (*) *p* < .05, (**) *p* < .01, (ns) non‐significant. Data were represented as the average ± *SEM*. Blue and black statistical characters indicate comparison of the responses to control and FGF2 conditions, respectively

Inhibition of Cx43 or P2Y1 receptor blocked FGF2‐induced proliferation in tanycytic cells in vitro. This suggests that FGF2 signaling might be upstream via the purinergic pathway and activated by auto‐ or paracrine molecules released through Cx43 HCs. Previous studies have shown that FGF1 and FGF2 affect the release of ATP to the extracellular medium, and that this occurs after 7 hr of exposure to the mitogen (Schalper et al., 2008). In the present work, the effect of FGF2 on the functional state of Cx43 HCs was studied. The sensitivity of the ethidium uptake (Etd^+^) to Gap27 and lanthanum (La^3+^), a specific Cx HC inhibitor, was evaluated in the presence of physiological concentrations of divalent cations as described previously (Schalper et al., 2008). Confluent tanycyte cultures were treated with 20 ng/ml FGF2, 10 IU/mL heparin, and/or 200 µM Gap27 for 7 hr. Etd^+^ uptake was evaluated by nuclear fluorescence intensity according to the protocol described in Figure 4a. The FGF2/heparin combination significantly increased the Etd^+^ nuclear fluorescence over time (around 700 AU on average at 25 min of recording; Figure 4b) compared to the control condition and with heparin (608.7 AU and 614 AU at the same time, respectively). Moreover, the FGF2/heparin‐induced Etd^+^ uptake was inhibited by La^3+^ (gray stripe in Figure 4b). Treatment with Gap27, even in the presence of FGF2/heparin, prevented this effect to values similar to the control conditions (607.6 AU at 25 min). The presence of the inhibitor itself had no effect (conjugated to heparin; 632.2 AU at 25 min), suggesting that Cx HCs do not play a relevant role under control conditions. For a more comprehensive visualization, only some treatments are shown in Figure 4b.

The Etd^+^ uptake rate was calculated as the slope of the curves shown in Figure 4b and allowed us to compare the sensitivity of the Etd^+^ uptake rate to different treatments (Figure 4c). Under normal conditions, the rate of Etd^+^ uptake by tanycytes presented a baseline value of 1.65 ± 0.10 AU/min (mean ± standard error), which decreased to 0.94 ± 0.08 AU/min after the addition of La^3+‐^_,_ which was not significant. After the addition of the FGF2 cofactor, heparin, the Etd^+^ uptake increased to 2.35 ± 0.50 AU/min although it was not significant. La^3+^ applied at 25 min of recording reduced the Etd^+^ uptake rate to a value similar to that of the control condition, La^3+^ (0.86 ± 0.14 AU/min). To determine the pathway through which FGF2 increases tanycyte membrane permeability, a pharmacological criterion was applied; the increase in the levels of uptake observed in the presence of the FGF2/heparin combination (4.70 ± 0.35 AU/min) was significantly reduced after the application of La^3+^ at 25 min of recording (0.71 ± 0.27 AU/min). In addition, values were restored to those similar to the control condition when incubated 7 hr with Gap27 (2.00 ± 0.64 AU/min and 0.58 ± 0.64 AU/min in the presence of La^3+^). The presence of Gap27 per se had no significant effect on the parameters evaluated with respect to the control (1.57 ± 0.52 AU/min and 0.40 ± 0.22 AU/min with La^3+^). The data confirm that tanycyte membrane permeability induced by treatment with FGF2 and measured at 7 hr, depends mainly on the activity of Cx43 HCs.

In primary cultures of cortical astrocytes, Cx43 HCs provide a pathway for the uptake and release of small molecules, including those involved in auto‐ and paracrine signaling, such as ATP (Garré et al., 2010). In order to explore whether the opening of Cx HCs induced by FGF2 contributes to the release of ATP, the concentration of ATP in the culture medium of tanycytes treated for 7 hr with FGF2 was evaluated using the luciferin luciferase assay (Figure 4d). In the presence of the FGF2/heparin complex, the concentration of ATP released to the medium and normalized to the protein concentration was 6.3 times greater than that obtained under control conditions (24.0 ± 2.0 pM/µg × µl^−1^; mean ± *SEM*) and 9.1 times more than that induced by heparin alone (16.6 ± 3.6 pM/µg × µl^−1^), reaching values of 152.6 ± 30.5 pM/µg × µl^−1^. However, incubation of FGF2/heparin with Gap27 significantly attenuated the ATP release to the extracellular medium, reaching average levels of 48.8 ± 21.1 pM/µg × µl^−1^. Again, the presence of the Gap27 inhibitor had no per se effect, and the residual ATP values remained close to the control (32.3 ± 6.1 pM/µg × µl^−1^).

### 3.4 Extracellular ATP exerts a mitogenic effect on cultured tanycytes

**FIGURE 5:**
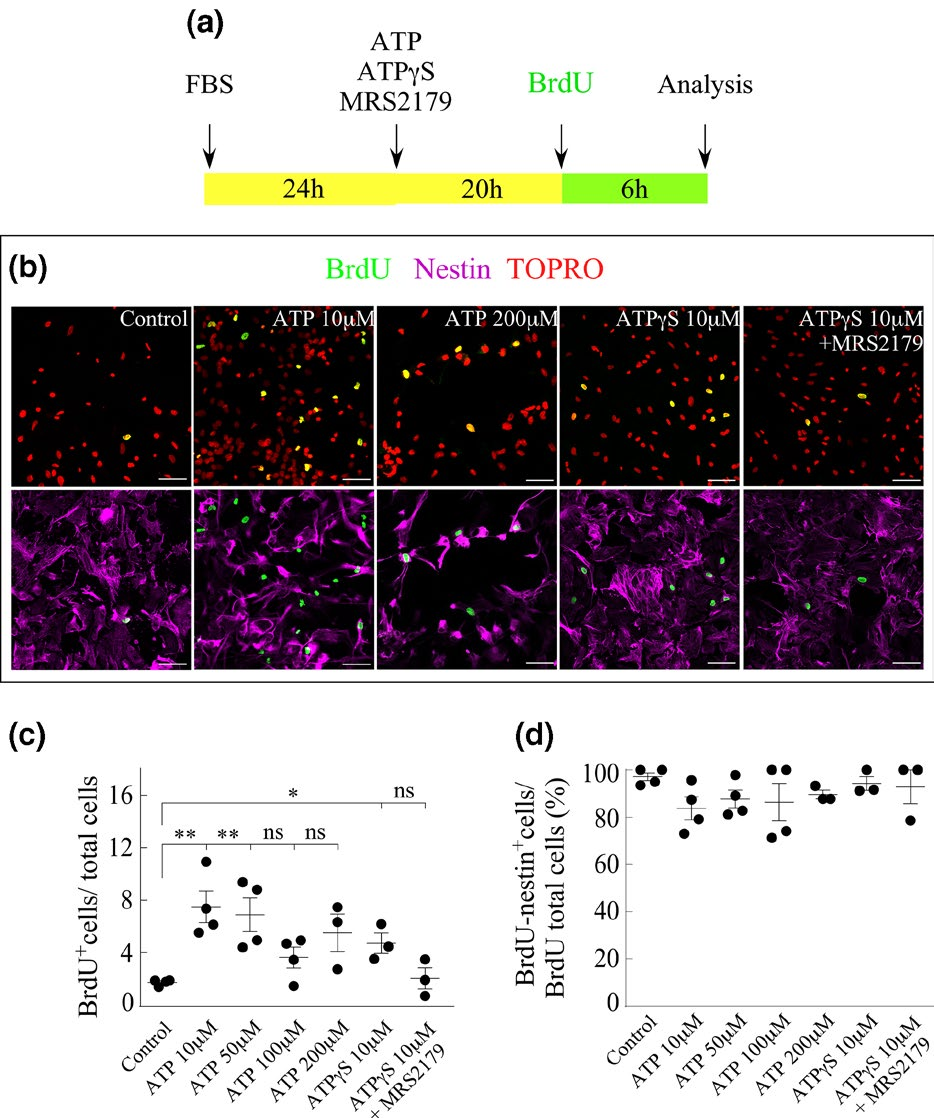
Proliferation of cultured tanycytes is promoted by ATP and ATPƔS and repressed by inhibition of P2Y1 receptors. (a) Timeline indicating when the procedures to evaluate BrdU incorporation were performed. (b) Immunofluorescence signal detected with antibodies reactive to BrdU (green) and nestin (magenta). TOPRO was used for nuclei staining (red). Scale bar: 100 µm. (c) Percentage of BrdU‐positive cells after exposure to 10 µM, 50 µM, 100 µM, 200 µM ATP, 10 µM ATPƔS, or 10 µM ATPƔS plus 10 µM MRS2179. (d) BrdU‐ and Nestin‐positive cells/BrdU total cells obtained in measurements of the amount of extracellular ATP (μM). *N*= ≥9 replicates and three independent cultures per condition. One‐way ANOVA with Bonferroni post hoc for control, 10 µM, 50 µM, 100 µM, and 200 µM ATP (first group analyzed) and control, 10 µM ATPƔS, and 10 µM ATPƔS plus 10 µM MRS2179 (second group independently analyzed). (*) *p* < .05, (**) *p* < .01, (ns) non‐significant. Data were represented as the average ± *SEM*

Previous studies have shown that extracellular ATP can exert a long‐term trophic effect in cultured astrocytes that includes promotion of DNA synthesis and cell division (Neary et al., 1998). It has been proposed that these events are mediated mainly by the activation of P2Y receptors (Neary et al., 1998). To investigate the possible mitogenic action of purinergic signaling activated by extracellular ATP, which can be released into the medium through Cx43 HCs once induced by FGF2, the incorporation of BrdU by tanycytes treated with the following conditions was assessed: (1) increasing ATP concentrations, (2) a non‐hydrolyzable analog of ATP, ATPƔS, and (3) ATPƔS in addition to MRS2179, a competitive P2Y1 receptor inhibitor. The different treatments were applied according to the protocol described in Figure 5a. Thus, we conducted concentration‐response type experiments, in which tanycytes were treated with increasing concentrations of ATP ranging from 10 to 200 µM. Immunocytochemistry assays were performed with anti‐BrdU (green) and‐nestin (magenta) specific antibodies, using TOPRO as the nuclear stain (red) (representative images in Figure 5b). The number of BrdU+cells/total cells (Figure 5c) and the number of double positive cells (BrdU+/nestin+) over the total number of BrdU+cells (Figure 5d) was quantified, respectively. The latter serves as a control to assess the tanycyte‐specific response to the stimuli. At 10 µM and 50 µM ATP, DNA synthesis in tanycytic cells was significantly higher than in cells under control conditions (Figure 5c), increasing the percentage of proliferative cells from 1.7 ± 0.1% (mean ± *SEM*) to 7.5 ± 1.2% (for 10 µM ATP) and 6.9 ± 1.3% (for 50 µM ATP). However, increasing concentrations of ATP did not lead to a concomitant increase in DNA synthesis, since at 100 µM and 200 µM, the percentage of transiting mitotic cells was 3.6 ± 0.8% and 5.5 ± 1.4%, respectively.

Two main classes of cell surface purinergic receptors have been described (Burnstock & Kennedy, 1985), which are the ATP P2 receptors and adenosine P1 receptors. The latter can be activated directly by adenosine or indirectly by products that result from the breakdown of ATP to adenosine by ectonucleotidases. To determine if the stimulation of cell division was because of the activation of P2 and/or P1 receptors in tanycytes, the hydrolysis‐resistant ATP analog, ATPƔS (Neary et al., 1998), and MRS2179, a P2Y1 receptor inhibitor, were used (Figure 5c). ATPƔS (10 µM) was sufficient to trigger a significant increase in tanycyte proliferation (4.7 ± 1.4%) compared to the control conditions, while 100 µM MRS2179 prevented it, reducing proliferation values closer to those of the control conditions (2.0 ± 0.8%). As described above, quantification of the double labeled nestin^+^/BrdU^+^ cells over the total of proliferating cells ranged from 83.8 ± 4.9 (for 10 mM ATP) to 97.1 ± 1.7% (for control), indicating that the observed response is cell type‐specific (Figure 5d). Along the experiments and conditions, cells expressed nestin and exhibited the typical expanded morphology of cultured tanycytes.

### 3.5 FGF2 positively modulates connexin43 expression in tanycytes at 7 hr

**FIGURE 6:**
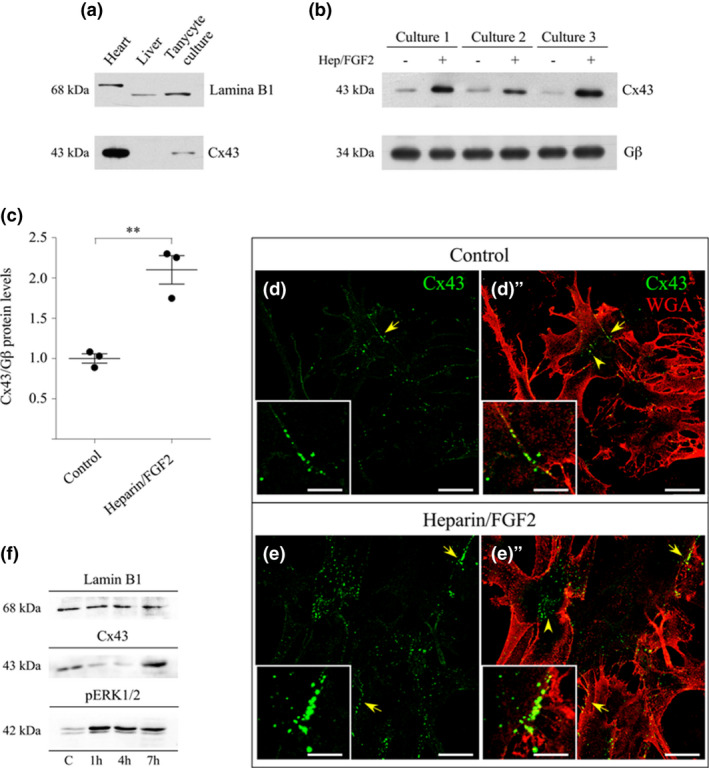
FGF2 increases the total amount of Cx43 in cultured tanycytes. (a) Specific Cx43 immunodetection in whole cardiac (positive control) and hepatic tissue (negative control), as well as of the total protein extraction of tanycyte primary culture. Lamin B1 was used as a loading control. (b) Cx43 detection by western blot analyses of three independent tanycytes cultures with and without exposure to 7 hr heparin/FGF2 treatment. The G protein β subunit was used as a loading control (Gβ). (c) Densitometric analysis of Cx43 after treatment with heparin/FGF2 with respect to Gβ and normalized to the control situation. *N* = 3 independent cultures for each condition. (*) *p* < .01, T‐test. Data were represented as the average ± *SEM*. Primary culture of tanycytes without treatment (d‐d’) or after 7 hr heparin/FGF2 induction (e‐e’) were fixed, and immunofluorescence analysis was performed using antibodies against Cx43 (green) and WGA (red). The yellow arrows point to Cx43 at the cell boundaries, co‐localizing with WGA. Arrowheads show the intracellular Cx43. Scale bar: 50 µm. The boxes show an amplification of the respective images in the region marked with the yellow arrows. Scale bar:

What mechanisms could explain the late involvement of Cx43HCs in the FGF2‐induced permeabilization? The results by far showed that treatment with FGF2 for 7 hr increased Cx43 HC activity and release of ATP to the extracellular medium. The half‐life of Cx43 is about 1.3 hr in cardiac tissue (Pogoda et al., 2016), suggesting that the detected changes in cell permeability mediated by Cx HCs could be a consequence of changes in Cx43 expression. To address this premise, the total amount of Cx43 after the FGF2 induction time was analyzed by immunoblots (Figure 6a–c and f) and immunofluorescence assays (Figure 6d‐e'). The specificity for the antibody used for Cx43 immunodetection was demonstrated using heart protein extract as a positive control and liver protein extract as a known tissue with very low expression (Figure 6a). Immunodetection assays were performed in protein extracts derived from three independent cultures (Figure 6b), which had approximately twofold increase in total Cx43 after 7 hr of heparin/FGF2 treatment (Figure 6c). The values were normalized to the G protein β subunit, which serves as a loading control for cell membrane proteins. For immunofluorescence assays to identify the cell membrane, the cells were fixed and briefly dipped with a solution containing WGA dissolved in PBS. WGA is a lectin that binds to the N‐acetyl‐D‐glucosamine and N‐acetylneuraminic acid monosaccharides and its derivatives (Acarin et al., 1994) and can be detected with anti‐WGA antibodies (in red). Co‐localization of anti‐Cx43 antibody binding sites with anti‐WGA was observed in the outer limits of the cell (Figure 6d‐d', arrows), indicating the presence of Cx43 on the cell membrane surface. Treatment with FGF2 and its cofactor induced an apparent increase in the amount of Cx43 content at the cell membrane and in the intracellular compartment with respect to the control conditions (Figure 6e‐e', arrows and arrowheads, respectively). A more detailed timeframe is shown in Figure 6f, where the amount of Cx43 decreased at 1 and 4 hr of FGF2/heparin treatment and then increased at 7 hr. To study whether these changes in Cx43 abundance were related to the FGF2 signaling pathway, the amount of phosphorylated ERK1/2 was analyzed at the same points (Figure 6f). The analysis showed an inverse relationship between the amount of Cx43 and the state of phosphorylated (p)ERK1/2 once treated with FGF2/heparin, the maximum of which occurred between hours 1 and 4 of activation by the ligand. These findings suggest that components of the FGF2 pathway regulate Cx43 through molecular events that were not discussed here, for example, by its the C‐terminal phosphorylation or the down‐regulation of its degradation (Axelsen et al., 2013).

In tanycytes, Cx43 HCs seem to have a fundamental role in glucose detection (Orellana et al., 2012) and in the activation of purinergic signaling induced by FGF2. However, in radial glia and adult NPs, gap junctions also participate in the cell cycle synchronization through the existence of a coupling network that permits the propagation of calcium waves (Weissman et al., 2004). Since tanycytes are robustly coupled to each other and to astrocytes and oligodendrocytes (Recabal et al., 2018), we wondered if cultured tanycytes retain the capacity to form coupling networks and if these are affected by FGF2. To address this question, tanycytes were grown on coverslips previously covered with poly‐l‐lysine up to 90% confluence and supplemented with serum‐free culture medium. Using a glass microelectrode (Figure S1a and b, asterisk), a single cell was filled with Lucifer yellow (Stewart & Wiley, 1981) for 5 min, then the spread of the molecule to other cells was observed under by fluorescence microscopy under control conditions (Figure S1a‐a') and after 7 hr of heparin/FGF2 treatment (Figure S1b‐b'). Under normal conditions, tanycytes established gap junctional communication in vitro forming clusters of at least two cells, although there were also more extensive configurations of 10 or more cells, reaching an average of nine coupled cells (Figure S1c). Heparin/FGF2 treatment induced the uncoupling of tanycytes to groups of ~3.2 cells on average (Figure S1d). These results suggest that FGF2 increases HC activity and reduces gap junctional communication between cells. Although these data are not statistically comparable, previous studies have shown the internalization and redistribution of Cx43 gap junction channels during cellular proliferation (Vanderpuye et al., 2016).

### 3.6 Gap27 inhibits FGF2‐induced proliferation of β‐tanycytes in vivo

**FIGURE 7:**
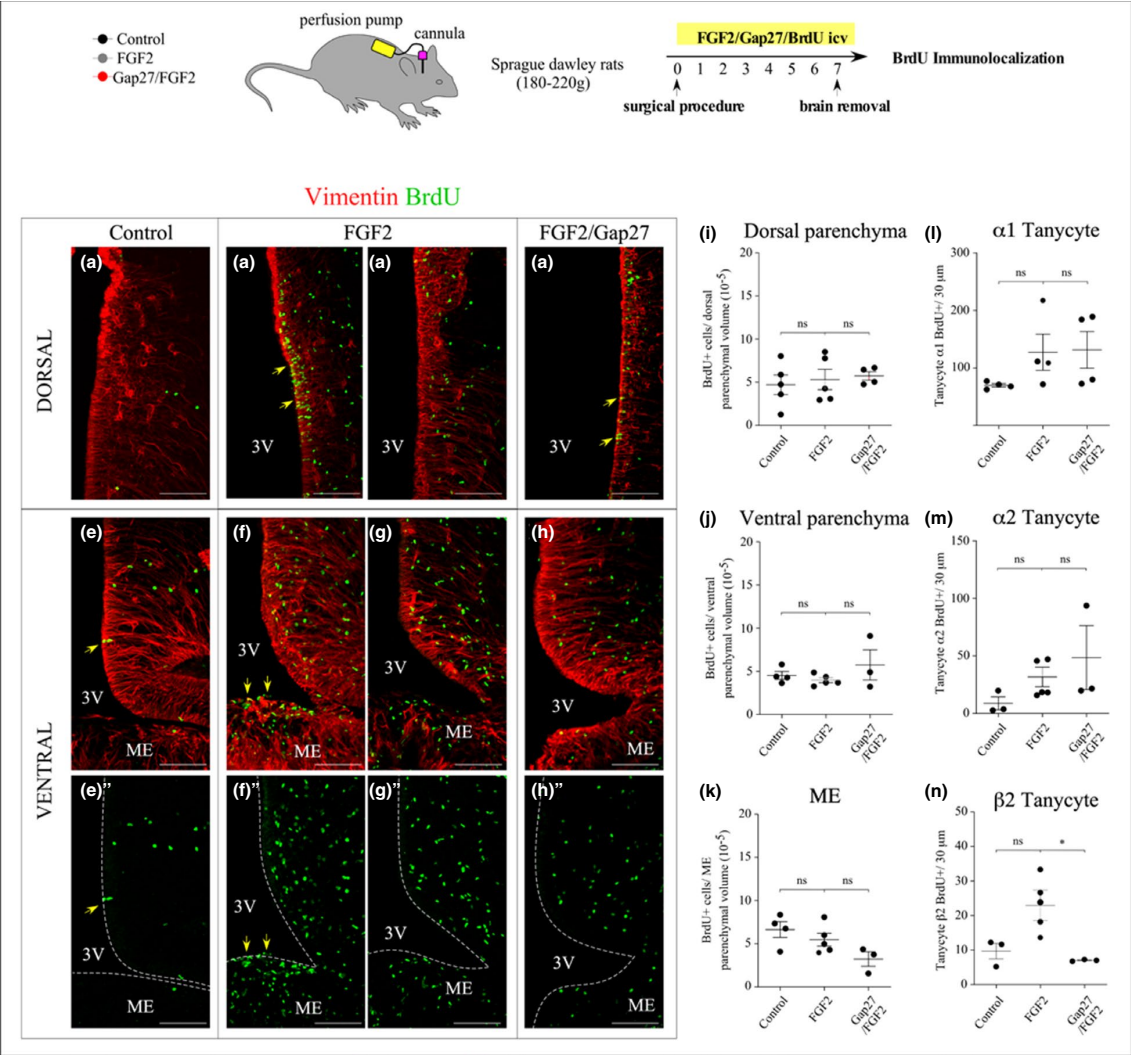
Proliferation of hypothalamic cells after ICV administration of FGF2 and Gap27. Immunohistochemistry of rat hypothalamic frontal sections when exposed for 7 consecutive days to vehicle and BrdU ICV infusion (a, e‐e'), FGF2 (b and c, f‐f’, g‐g'), and FGF2/Gap27 (d, h‐h'). Using specific antibodies, the incorporation of BrdU (green) by hypothalamic cells, including vimentin‐labeled tanycytes (red), are shown. (a–d) Dorsal hypothalamic portion, which includes the subpopulation of α1‐tanycytes and ependymocytes. (e‐h’) Ventral hypothalamic portion, considering α2, β1, and β2‐tanycytes, in addition to ME cells. The yellow arrows indicate the incorporation of BrdU by tanycytes. Scale bar: 100 µm. 3V, third ventricle. ME, Median Eminence. (i–n) Number of BrdU‐positive cells normalized to tissue volume in the parenchyma of the dorsal (i), ventral (j), and ME (j) hypothalamus. (l–n) Proliferation of tanycyte subpopulations normalized to 30 µm slice thickness; (l) α1‐tanycytes and ependymocytes, (m) α2‐tanycytes, and (n) β2‐tanycytes. *N* = at least 3 animals and 31 slices for each condition. One way ANOVA. (*) *p* < .05, (ns) not significant. Data are represented as mean ± *SEM*

In order to detect changes in hypothalamic cell turnover upon FGF2 and Gap27 treatment in vivo, the cell proliferation assay was performed by continuous release of BrdU into the 3V. Osmotic pumps (Alzet, model 1007D) connected to the ventricular brain system by a cannula to continuously deliver FGF2 and Gap27 for 7 days at a rate of 0.0125 µg/h and 0.13 µg/h, respectively. BrdU (0.75 µg/h) was co‐administered to verify stimulation or interruption of cell proliferation, as shown in the schematic representation of Figure 7. The rats were sacrificed 8 days after the implantation of the osmotic pump and the brains were removed for subsequent immunohistochemical analysis through the co‐localization of BrdU (green) with vimentin, a tanycyte marker (red) (Figure 7). The cell division representative of the control situation (BrdU infusion only, Figure 7a and e‐e'), after exposure to FGF2 (Figure 7b and c and f‐g') and FGF2/Gap27 (D‐H') were detailed in the dorsal hypothalamic portion (Figure 7a–d) and in the ventral portion (Figure 7e‐h'). The BrdU positive ventricular cells, conceivably tanycytes, from both the lateral ventricular wall and the floor of the 3V are denoted with yellow arrows in Figure 7b, d and e‐f’. The infusion with FGF2 induced an increase in the number of highly variable proliferative ventricular cells, with sections with great incorporation of BrdU in the lateral VZ (Figure 7b) and ventral (Figure 7f‐f') and others with scarce BrdU labeling in the same areas (Figure 7c and g‐g', respectively). The dispersion of these data is evident in Figure 7l.

The quantitative analysis of BrdU‐positive (BrdU+) cells was computationally performed, considering the number of parenchymal and ventricular cells labeled, both for the dorsal and ventral sections and for the Median Eminence (ME) (Figure 7i–n). All the data are represented as mean ± *SEM*. The number of proliferative parenchymal cells in the dorsal hypothalamus of control animals was 4.7 × 10^−5^ ± 1.1 × 10^−5^ BrdU+cells/per µm^3^ of tissue (Figure 7i), although FGF2 and FGF2/Gap27 treatment groups did not vary significantly compared to the control (5.3 × 10^−5^ ± 1.2 × 10^−5^ and 5.7 × 10^−5^ ± 4.8 × 10^−6^ BrdU+cells/µm^3^, respectively). The number of cells that incorporated BrdU in the ventral hypothalami did not vary significantly between the control, FGF2, and FGF2/Gap27 conditions, being 4.5 × 10^−5^ ± 4.6 × 10^−6^, 4.0 × 10^−5^ ± 2.8 × 10^−6^, and 5.7 × 10^−5^ ± 1.7 × 10^−5^ BrdU+cells/µm^3^ for each group, respectively (Figure 7j). Neither in the ME were significant changes in BrdU incorporation, being the counts 6.6 × 10^−5^ ± 9.1 × 10^−6^, 5.5 × 10^−5^ ± 7.4 × 10^−6^, and 3.2 × 10^−5^ ± 8.5 × 10^−6^ BrdU + cells/µm^3^ for control, FGF2, and FGF2/Gap27, respectively (Figure 7k). Next, the number of ventricular cells that underwent proliferation was quantified, defining them as tanycytes and/or ependymocytes if their nuclei were located up to 20 µm apart from the ventricular wall. For the count, the following population were considered: α1‐tanycytes and ependymocytes (Figure 7l), α2‐tanycytes (Figure 7m), and β2‐tanycytes (Figure 7n). The number of all the ventricular cells mentioned was normalized to 30 µm tissue thickness. Robins et al. (2013) showed that FGF2 stimulates the proliferation of α2‐tanycytes, which explains why the infusion of this mitogen was used as a positive control in our procedure. The α1‐tanycytes and ependymocytes showed a basal proliferation of 70 ± 3 BrdU+cells/ 30 µm thickness (Figure 7l), which increased, although not significantly, in the presence of FGF2 (127.3 ± 31.4 BrdU+cells/30 µm tissue thickness). Curiously, the infusion of both compounds (FGF2/ Gap27) did not significantly decrease the proliferation of α1‐tanycytes and ependymocytes with respect to that induced by FGF2 nor to the control (Figure 7d, 131.4 ± 31.9 BrdU+cells/30 µm tissue thickness, respectively). Although with a smaller number of cells, a similar effect was observed for α2‐tanycytes (Figure 7m), whose basal proliferation (8.7 ± 5.6 BrdU+cells/30 µm tissue thickness) increased, but not significantly, in the presence of FGF2 (31.7±8.5 BrdU+cells/ 30 µm tissue tickness) and this could not be reversed in the presence of Gap27 (48.6 ± 27.8 BrdU + cells/ 30 µm tissue thickness). The quantitative analysis of β2‐tanycytes proliferation revealed an increase, although not significant (Bonferroni post hoc analysis), in their cell division after induction with FGF2 (23 ± 4.4 BrdU+cells/ 30 µm tissue thickness) compared to the control (9.7 ± 2.2 BrdU+cells/ 30 µm tissue thickness), which could be significantly blocked by Gap27 (7.0 ± 0.1 BrdU+cells/30 µm tissue thickness; Figure 7n). Thus, β2‐tanycytes were the only cell type affected by Cx43 inhibition, which was required for the FGF2‐induced cell division. Finally, our in vitro results agreed with those in vivo, indirectly evincing the preferentially β subpopulation content in the tanycyte cultures.

## 4 DISCUSSION

Previous studies on Cx43 gap junctions in ex vivo tanycytes showed that they are robustly coupled to each other (Szilvasy‐Szabo et al., 2017) and to astrocytes and oligodendrocytes (Recabal et al., 2018). Moreover, Cx43 was the only connexin responsible for tanycyte coupling network. In the present work, the role of Cx43 as HCs on tanycytes was explored. However, we cannot exclude that some of the effects seen, that is, in cell division, were in part due gap junction blockade. Inhibition of Cx43 HCs in tanycytes prevented FGF2‐induced proliferation in vitro and in vivo, suggesting the involvement of both proteins in a common pathway. In C6 glioma cells (De Vuyst et al., 2007) and HeLa cells (Schalper et al., 2008) transfected with Cx43, as well as in spinal astrocytes (Garré et al., 2010), FGF1 or FGF2 induces a transient opening of the formed Cx43 HCs, through which ATP is released. Etd^+^ uptake analysis revealed that FGF2 promotes the activity of tanycytic Cx43 HCs, which were partially but drastically inhibited by Gap27, suggesting that this connexin is primarily responsible for the dye uptake. However, the contribution of other connexins (such as Cx45, highly expressed in tanycyte cultures) and pannexins (particularly Panx1 and Panx2) (Recabal et al., 2018), which allow the diffusion of molecules across the cell membrane, cannot be ruled out. Opening of Cx43 HCs induced by FGF2 led to the release of ~6.3‐fold more ATP compared to the control conditions. Although these values are low compared to the ~45‐fold increase of nucleotide release after treatment of tanycytes with 10 mM glucose (Orellana et al., 2012), it is important to consider the timing at which maximal response was elicited by cells treated with FGF2 (7 hr) and glucose (< 1 min). ATP output was significant, but partially inhibited by Gap27, suggesting that in addition to Cx43 HCs, other routes might be involved. It is known that the ATP release induced upon activation of FGFRs may be because of (1) vesicular release; (2) activation of P2X7 receptors, which in turn stimulate the opening of Panx1 channels, and (3) Cx43 HCs (Garré et al., 2010). However, Panx1 channels are not responsible for the ATP release induced by 10 mM glucose in tanycytes (Orellana et al., 2012).

In cultured tanycytes, we found that FGF2 increased the activity of Cx43 HCs as evaluated by Etd^+^ uptake in addition to ATP release assays. Previous studies performed in Cx43‐transfected HeLa cells showed that FGF1 increases the activity Cx43 HCs, which is associated with an increase in cell surface distribution rather than changes in the total amount of Cx43 (Schalper et al., 2008; De Vuyst et al., 2007). However, 10 mM glucose increases the ion flux through Cx43 HCs but does not affect the amount of Cx43 on the cell surface (Orellana et al., 2012). The modifications indicated are attributable to the phosphorylation of the Cx43 carboxy‐terminal domain by various kinases, including MAPK, which underlies the regulation of Cx43 HC opening and Cx43 distribution and degradation as well (Pogoda et al., 2016). The present work showed that 7 hr of FGF2 treatment doubled the total amount of Cx43 in tanycytes, an event that might be mediated by an ERK1/2‐dependent mechanism. In support to this statement, ERK1/2 phosphorylation was apparently inversely related to the amount of Cx43 throughout the FGF2 treatment. Nevertheless, the amount of Cx43 correlates positively with ERK1/2‐dependent phosphorylation in endothelial cells (Arshad et al., 2018). It is also possible that FGF2 signaling reduces the degradation instead of increasing expression and synthesis of Cx43 (Axelsen et al., 2013). Hence, the molecular mechanism by which FGF2 regulates the amount of Cx43 and opening of Cx43 HCs in tanycytes requires further studies.

**FIGURE 8:**
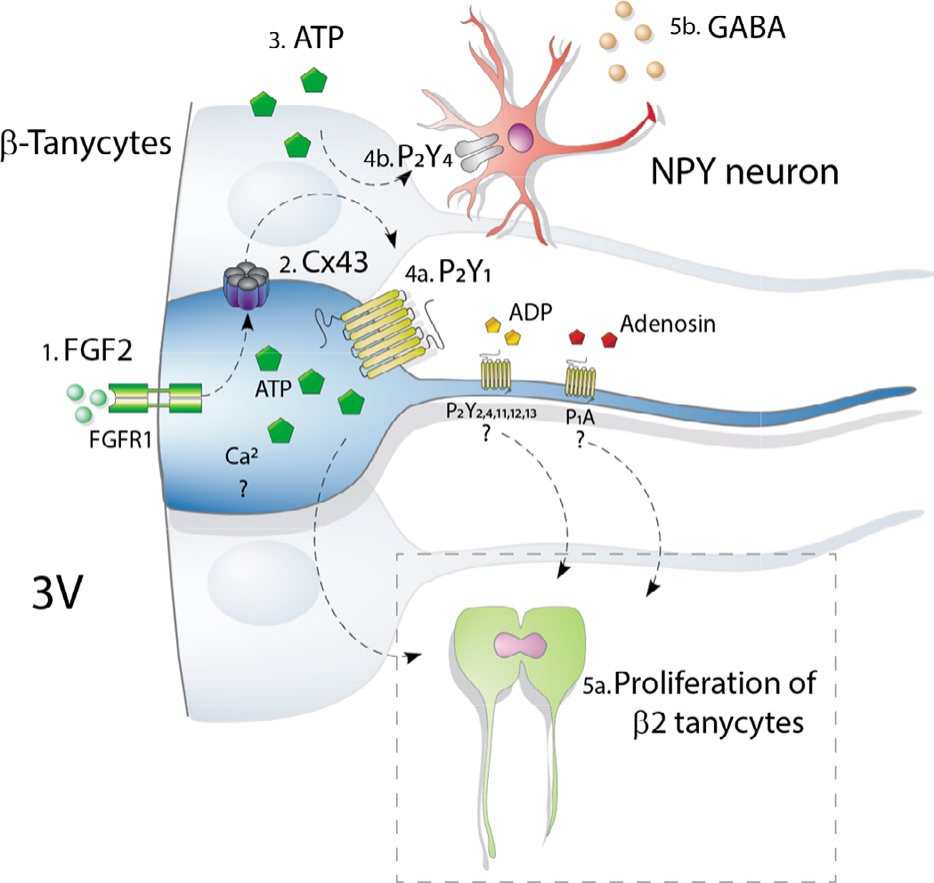
Scheme representing the effect of FGF2 on the activity of connexin hemichannels and consequences on ATP release and proliferation of cultured tanycytes. 1) FGF2 induces proliferation of cultured tanycytes via activation of FGFR1, leading to 2) increase connexin43 hemichannel (Cx43 HC) opening, and 3) ATP release via Cx43 HCs. ATP through autocrine signaling could activate 4a) P2Y1 located in the tanycytes for inducing [Ca^2+^]_i_ increases, which could mediate 5a) tanycyte proliferation. In addition, P2Y 2,4,11,12, and 13 receptors located in tanycytes and the adenosine receptor P1A could potentiate these effects. Another action that could be attributed to ATP released by the tanycytes, is the 4b) paracrine activation of P2X4R in NPY neurons that release GABA

ATP release can exert long‐term effects, such as proliferation, differentiation, migration, and apoptosis in various cell types, especially in astrocytes (Neary et al., 1998) and embryonic NPs (Weissman et al., 2004). The current work showed that tanycytes increased their cell division when they were exposed to 10 or 50 µM ATP, with concentrations greater than 100 µM having no significant effects. The concentration‐dependent effect agrees with studies in astrocytes (Neary et al., 2008) and NPs of the adult subventricular zone (ZSV) (Mishra et al., 2006), where ~30–50 µM of this nucleotide potentiates FGF2‐induced proliferation and 300 and 1,000 µM inhibit it (Neary et al., 2008). Low and high concentrations of ATP activate P2Y and P2X receptors, respectively, triggering opposite effects in the cells, while activation of P2Y receptors promotes DNA synthesis and activation of P2X7 receptors induces arrest of the astrocyte cell cycle in a resting state (Neary et al., 2008). On the other hand, the expression of hypothalamic P2X4 receptors (ATP ionotropic receptors) is limited to NPY orexygenic neurons and tanycytes (Xu et al., 2016). The functional role of this receptor type in NPY neurons, once activated by ATP, is to facilitate the release of GABA from the pre‐synaptic terminal on the two post‐synaptic targets—ARC POMC anorexigenic neurons and paraventricular nucleus neurons (Xu et al., 2016). Although it is known that under sugary (Frayling et al., 2011) and different sweetener stimuli (Benford et al., 2017), tanycytes undergo activation of a purinergic P2 receptor signaling, and the functional role of P2X4 receptors in tanycytes remains to be elucidated. It is reasonable to propose that FGF2‐induced release of ATP by tanycytes has an impact not only autocrine proliferative through P2Y1 receptors, but it might also be paracrine, through the activation of P2X4 receptors in NPY neurons, as is depicted in the final scheme (Figure 8).

The eight P2Y receptor subtypes can be activated by ATP, ADP, UTP, UDP, and nucleosides (Figure 8), and are linked to different signaling cascades (Zimmermann, 2006). The presence of ectonucleotidase enzymes, whose catalytic site is facing the cell exterior, controls the functionality of extracellular nucleotides. Specifically, the nucleoside triphosphate diphosphohydrolase 2 (ENTPDase2) enzyme is highly expressed by PNs of the adult neurogenic niches, the subventricular and subgranular zones, and catalyzes the hydrolysis of nucleoside triphosphates, transforming them into di and subsequently, monophosphate (Gampe et al., 2015; Mishra et al., 2006). RNAseq studies in cultured tanycytes (Recabal et al., 2018) indicate that the ENTPDase2 transcripts are highly represented, suggesting that the proliferation observed after their exposure to ATP could be a consequence of the molecular interactions that result from ATP degradation (ADP, AMP, and adenosine) with the P2Y1,2,4,11,12,13 and/or P1 receptors (Zimmermann, 2006). To circumvent the lack of specificity, the non‐hydrolyzable analog of ATP, ATPƔS, and the specific inhibitor of P2Y1, and MRS2179, were used. This approach demonstrated that ATPƔS is sufficient to induce an increase in BrdU incorporation by tanycytes, a response that was blocked by MRS2179, suggesting that the hydrolysis of ATP by ectonucleotidases was not essential to achieve the proliferative effect. However, because the quantification of BrdU incorporated upon treatment with 10 mM ATP was slightly higher than that induced with the same concentration of ATPƔS, it is not possible to completely rule out the participation of ADP and adenosine activating other purinergic receptors. Our data coincide with those provided by the literature for the SVZ NPs, which present high hemichannel activity (Talaverón et al., 2015), through which ATP could be mobilized to the outside of the cell to promote cell proliferation once the purinergic receptors are activated (Suyama et al., 2012). Inhibition of either ATP release or activation of purinergic receptor affected cell proliferation. Figure 8 shows another action that could be attributed to ATP released by the tanycytes. Xu et al. (2016) have shown that ATP could mediate activation of P2X4R in NPY neurons that release GABA for inhibiting POMC neurons and, therefore, contribute to regulating feeding behavior. The effects entailed by purine release from tanycytes are physiologically broad, both in a long and short term, ranging from promoting cellular division to triggering activation of neuronal orexigenic responses.

In the present work, it was demonstrated that Gap27, a Cx43HC inhibitor, reduced the FGF2‐induced proliferation of β2‐tanycytes, which have been controversially proposed as PNs of the adult hypothalamus (Kano et al., 2019; Lee & Blackshaw, 2012). This proliferative blockade seems to be unique to this cell subtype, since parenchymal cells of hypothalamic neuronal nuclei, ME, and α‐tanycyte cells did not show such dramatic decrease in BrdU incorporation upon FGF2 treatment. Notably, similar unalterable proliferative state of parenchymal cells in the ARC has been previously observed after HFD treatment (Safahani et al., 2019). Studies exploring changes in the metabolic state of the individual support the conception of ME‐residing cells plasticity. Clear examples of this are the morphological changes of the external ME area to facilitate the release of the gonadotropin‐releasing hormone into the portal circulation during the estrous cycle, which consists of a close up of the parenchymal basal lamina to the neuronal terminals (Prevot et al., 1999). Consistent with this, female rats treated with a HFD show increased proliferation and neurogenesis specifically in ME (Lee et al., 2014). Previously, the same researchers elucidated the origin of adult nascent neurons, attributing it to β2‐tanycytes (Lee et al., 2012). Hence, Cx43 HCs plays a crucial role not only in the detection of glucose by tanycytes (Frayling et al., 2011; Orellana et al., 2012), but also in the self‐renewal of β2‐tanycytes, evidencing the versatility of membrane channel. The tanycyte multiplication likely depends on the concentration of FGF2 and Gap27 reached in the CSF, which may have not been optimal to achieve noticeable responses, for example, on the incorporation of BrdU by α‐tanycytes. This possibility remains to be further studied using higher FGF2 concentrations up to a detectable cell proliferation response that could be evaluated using a direct approach, for example through Ki67 measurements. In parallel the use of Gap27 or other selective Cx43 HC blocker would unveil the involvement of Cx43 HCs in FGF2‐induced cell proliferation.

Neurogenesis in adult life remains a controversial topic within the scientific community (Sorrells et al., 2018). In the last decade, hypothalamic neurogenesis has been proposed as an adaptive response mechanism to nutritional imbalance (Sousa‐Ferreira et al., 2014). Therefore, our results provide a novel mechanism involved in this process. Purinergic signaling is mediated in part by Cx43 HCs (and likely scattered by Cx43 gap junctions) that participate in the proliferation of hypothalamic tanycytes, a mechanism that could underlie the development of pharmacological approaches to regulate body weight and decrease the incidence of obesity.

## CONFLICT OF INTEREST

The authors declare that they have no competing interests.

## AUTHORS’ CONTRIBUTIONS

The experiments were performed at the Department of Cell Biology at the University of Concepcion and Departamento de Fisiología, Facultad de Ciencias Biológicas, Pontificia Universidad Católica de Chile, Santiago. MA.G‐R, A.R, and JC.S conceived the experiments; MA.G‐R., A.R., T.C, and JC.S designed the experiments; A.R, P.F., S.L., P.O, A.P, MJ.B, performed the experiments; A.R., P.O., C.F., R.E‐V. analyzed the data; MA.G‐R, A.R., C.F., T.C., E.U., and JC.S. contributed reagents/materials/analysis tools; MA.G‐R., A.R., and JC.S. wrote the paper, and critically revised the manuscript. All authors have approved the final version of the manuscript and agree to be accountable for all aspects of the work in ensuring that questions related to the accuracy or integrity of any part of the work are appropriately investigated and resolved. All persons designated as authors qualify for authorship, and all those who qualify for authorship are listed.

## Supporting information

Supplementary Material
