# Supplementary material for: The FGF2‐induced tanycyte proliferation involves a connexin 43 hemichannel/purinergic‐dependent pathway
Source: J Neurochem. 2020 Oct 19;156(2):182–99. doi: 10.1111/jnc.15188 (PMC7894481; doi:10.1111/jnc.15188)
Supplement: Supplementary file 1 — Supplementary Material [file JNC-156-182-s001.pdf]

The FGF2-induced tanycyte proliferation involves a connexin 43-hemichannel/purinergic pathway

Antonia Recabal[1,4], Paola Fernández[2], Sergio López[1], María José Barahona[1], Patricio Ordenes[1], Alejandra Palma[1], Roberto Elizondo-Vega[1], Carlos Farkas[5], Elena Uribe[1], Teresa Caprile[1], Juan C. Sáez[2,3*] and Maria A. García-Robles[1*]

[1]Departamento de Biología Celular, Universidad de Concepción, Concepción, Chile

[2]Departamento de Fisiología, Facultad de Ciencias Biológicas, Pontificia Universidad Católica de Chile, Santiago

[3]Instituto de Neurociencias, Centro Interdisciplinario de Neurociencias de Valparaíso, Universidad de Valparaíso, Valparaíso, Chile

[4]Current affiliation: Department of Physiology and Pathophysiology, Max Rady College of Medicine, University of Manitoba, Winnipeg, Manitoba, Canada

[5]Research Institute in Oncology and Hematology, Cancer Care Manitoba, Winnipeg, Manitoba, Canada

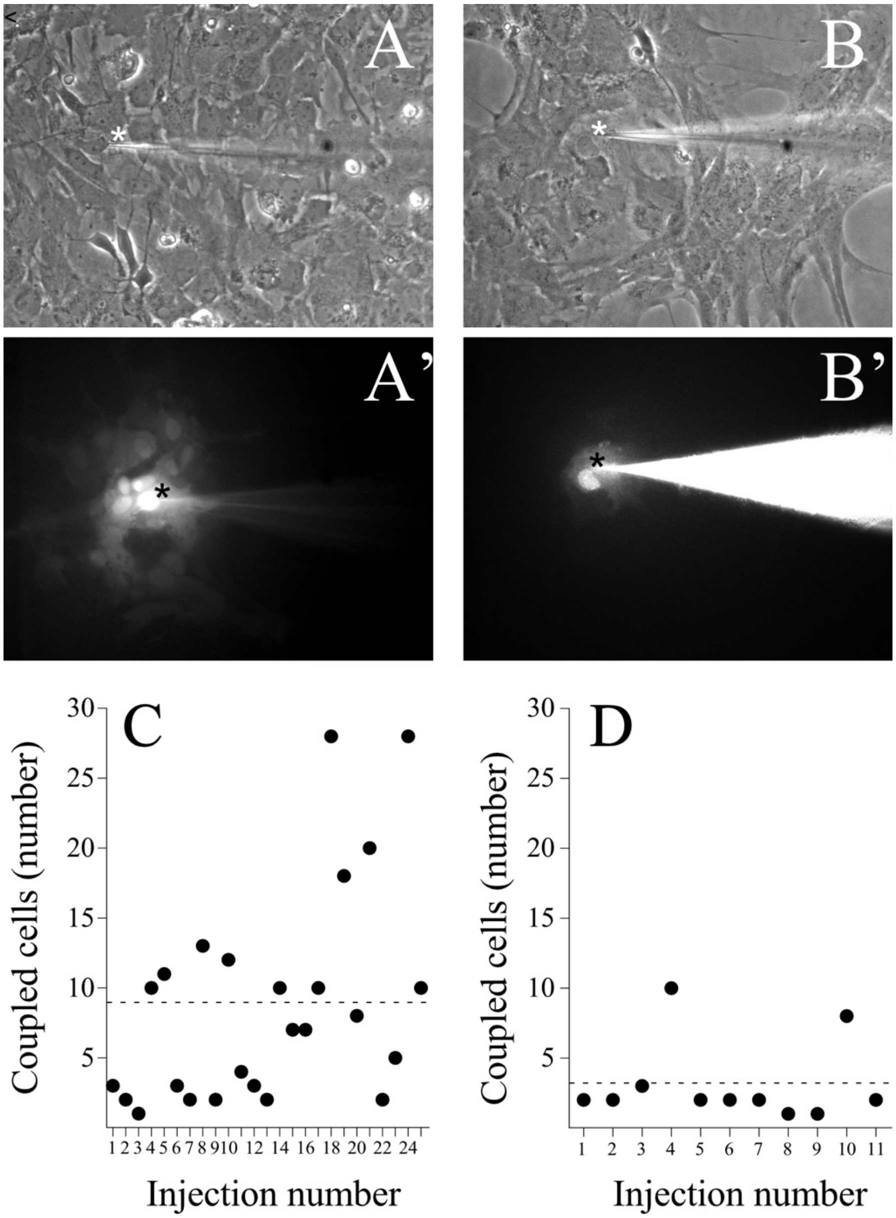

Figure Supplementary 1. FGF2 induces gap junctional uncoupling in cultured tanycytes.

Confluent tanycyte cultures were mounted on coverslips. One individual cell was visualized by bright field microscopy (A-B) and injected during 5 min with Lucifer yellow using a glass pipette (asterisk). This time frame allowed visualization of the diffusion of the fluorescent signal representative of Lucifer yellow to spread to adjacent

cells (A'-B '), indicating the conformation of an extensive network of more than 10 cells for the control condition (A, A') and about only 2 cells coupled to the heparin/FGF2 condition (B, B '). (C and D) Quantification of the number of coupled cells after each injection for the control (C) and heparin/FGF2 (D) condition. The dotted line defines the average of coupled cells, being 9.0 in (C) and 3.2 in (D). Data correspond to a triplicate of a single culture under each condition.
